# Reply to Keles, F. Beyond Systemic Inflammation: The Complementary Role of Cellular Stress Markers in Predicting STEMI Outcome. Comment on “Magureanu et al. Early Inflammatory Biomarkers, Ventricular Dysfunction and In-Hospital Mortality in Patients with ST-Elevation Myocardial Infarction Undergoing Primary Percutaneous Coronary Intervention. *Diagnostics* 2026, *16*, 1978”

**DOI:** 10.3390/diagnostics16172874

**Published:** 2026-09-07

**Authors:** Dan Claudiu Magureanu, Maria Luiza Hiceag, Camelia Bianca Rus, Timea Claudia Ghitea, Corina Cinezan

**Affiliations:** 1 Department of Pharmacology, Toxicology and Clinical Pharmacology, Iuliu Hatieganu University of Medicine and Pharmacy, 400012 Cluj-Napoca, Romania; 2 Cardiology Department, Niculae Stancioiu Heart Institute, 400001 Cluj-Napoca, Romania; 3 Cardiology Department, Rehabilitation Hospital, 400347 Cluj-Napoca, Romania; 4 Cardiology Department, Municipal Hospital Aiud, 515200 Aiud, Romania; 5 Department of Medical Disciplines, Faculty of Medicine and Pharmacy, University of Oradea, 410073 Oradea, Romania; 6 Clinical County Emergency Hospital Bihor, 410169 Oradea, Romania; 7 Doctoral School of Biological and Biomedical Sciences, University of Oradea, 410087 Oradea, Romania; 8 Pharmacy Department, Faculty of Medicine and Pharmacy, University of Oradea, 410073 Oradea, Romania

We sincerely thank the author for the thoughtful and constructive comment highlighting the potential complementary role of cellular stress markers in the prognostic assessment of patients with ST-segment elevation myocardial infarction (STEMI) [1].

We agree that the pathophysiological response to acute myocardial infarction extends beyond systemic inflammation and includes cellular stress, endothelial dysfunction, and myocardial injury. In this context, heat shock protein 60 (HSP60) represents an interesting potential biomarker of cellular stress. Previous evidence has demonstrated an association between elevated HSP60 levels and adverse in-hospital and short-term outcomes in patients with acute STEMI, including heart failure, cardiogenic shock, and mortality [2,3].

Mechanistically, systemic inflammatory biomarkers such as the neutrophil-to-lymphocyte ratio (NLR), the platelet-to-lymphocyte ratio (PLR), and C-reactive protein (CRP) reflect the systemic inflammatory response, whereas cellular stress markers such as HSP60 may provide complementary information regarding myocardial and cellular stress. These pathways are closely interconnected during acute myocardial infarction but may reflect different aspects of the pathophysiological response. Therefore, their combined assessment may potentially improve the early identification of patients at increased risk of hemodynamic deterioration.

Our study was specifically designed to investigate the prognostic relevance of early inflammatory biomarkers, including NLR, PLR, and CRP, in relation to ventricular dysfunction and in-hospital mortality following primary percutaneous coronary intervention. HSP60 and other cellular stress markers were not assessed in our cohort; therefore, their incremental prognostic value could not be evaluated in the present study.

Nevertheless, we consider the proposed integration of inflammatory and cellular stress biomarkers particularly relevant. Prospective studies incorporating HSP60 alongside established inflammatory indices, cardiac biomarkers, and clinical parameters would be valuable to determine whether such an integrated multimarker model provides incremental prognostic information beyond conventional risk stratification.

We appreciate this important suggestion, which highlights a promising direction for future research in the early risk stratification of patients with STEMI.

## Data Availability

No new data were created or analyzed in this study. Data sharing is not applicable to this article.
